# Improving the cancer adult patient support network (iCAN): a pilot study on a communication model and modified focus group

**DOI:** 10.3389/fpsyg.2023.1223168

**Published:** 2023-10-23

**Authors:** Giuseppina Campisi, Monica Bazzano, Rodolfo Mauceri, Vera Panzarella, Gaetano La Mantia, Olga Di Fede

**Affiliations:** ^1^Unit of Oral Medicine and Dentistry for Fragile Patients, Department of Rehabilitation, Fragility, and Continuity of Care, University Hospital Palermo, Palermo, Italy; ^2^Department Di.Chir.On.S., University of Palermo, Palermo, Italy; ^3^Department of Biomedical and Dental Sciences and Morphofunctional Imaging, The University of Messina, Messina, Italy

**Keywords:** oral medicine, focus group, cancer, communication, pain, dental hygiene, cancer treatments, empowering patients

## Abstract

**Background:**

Many consider that cancer has the greatest impact of any disease in the world, and it can drastically limit patients’ quality of life. Combating such a life-threatening disease can pose many challenges to daily life, highlighted by demonstrating the need to discuss one’s health status within a focus group and encourage treatment compliance.

**Aim:**

the purposes of this study were to share the authors’ experience of a modified focus group in an Oral Medicine Unit, termed “Improving Cancer Adult Patients Support Network” (iCAN), and to evaluate how effective communication could improve patients’ quality of life and empower them by virtue of enhanced knowledge and an awareness of cancer management.

**Methods:**

the paper adhered to the COREQ checklist regarding its reporting procedures. The iCAN format was precisely reproduced four times with four groups, consisting of 12 adult male and female patients with solid cancers. They discussed several main topics relating to cancer treatment, as chosen by a majority of the participants. Four specialists were involved in the discussion of the selected topics The iCAN format was faithfully reproduced during each meeting, with the participants in the roles of moderator and health specialists. Finally, a satisfaction questionnaire was administered.

**Results:**

the most reliable results demonstrated a marked change in lifestyle and eating habits in more than 50% of participants. More than 80% were unaware of the side effects of cancer treatments in general and the oral mucosa in particular. Each meeting reported a maximum degree of satisfaction experienced by the participants.

**Conclusion:**

iCAN focus group meetings appear to have facilitated a process of narrative interviewing, thereby improving the doctor-patient relationship underlying the humanization of the care process.

## 1. Introduction

A focus group can be defined as a research methodology for acquiring data during semi-structured discussions with groups of 4–12 people, which is dedicated to a specific topic (Sofaer, 2002). It is in essence a qualitative study whose data are not purely numerical, but it also concerns emotions, feelings, and the ideas of the people involved, regarding the topic under discussion. Focus groups can transfer new knowledge to and between patients and provide fresh perspectives on healthcare (Tong et al., 2007). Their use has become widespread in assessing the experience and awareness of being ill (Kitzinger, 1994; Wong, 2008). The recruitment of participants to a focus group is generally based on their experience and condition in relation to the research topic, having defined a statement of purpose. Interaction during the focus group sessions, as managed by the moderator, encourages participants to share individual experiences and communicate with each other, thereby exchanging ideas and comments on their experiences or views. Unlike one-on-one interviews, such group interaction can offer an added dimension of interactions among members: it is conducted in a precise and timely manner by the moderator who merely asks questions. Where there are reluctant participants, it is the moderator’s responsibility to formulate questions to engage theses participants (Kitzinger, 1995; Wong, 2008).

Thus, the focus group provides a platform for collecting and analyzing the experiences, thoughts, and emotions of patients, allowing for a comprehensive understanding of how cancer impacts their lives. Recent findings from a pilot study involving 279 participants assessing quality of life revealed a significant reduction in the quality of life for over 50% of the investigated sample (Alam et al., 2020).

There exist several studies relating to the focus groups of women with breast cancer, which have reported positive results (Ruddy et al., 2013; Kim et al., 2020a,b; Rafn et al., 2020; Nyrop et al., 2021; Schifferdecker et al., 2021). However, to the best of our acknowledgment, the experience of a focus group of male and female solid cancer patients has yet to be published.

## 2. Aim

The purposes of this study were to create a cancer focus group, named iCAN (an acronym of the improving Cancer Adult patients support Network), and to evaluate if it was capable of empowering patients with improved knowledge and awareness in managing various special healthcare needs.

## 3. Materials and methods

### 3.1. Study design

The study was approved by the local Ethical Committee (approval number 4/2015). Forty-eight solid cancer participants, who were being followed up for oral disorders or complications during/after cancer management, were recruited in a community hospital setting at the Oral Medicine and Dentistry Unit for frail patients (Palermo, Italy). These forty-eight patients comprised four cancer focus groups, and they were managed with the same iCAN format.

Each participant expressed an interest in participating in the research study and no one declined to participate or left the group. Researchers informed patients regarding the workings of the focus group with comprehensive information. This encompassed a detailed overview of the purpose, structure, and objectives of the focus group discussions. The patients were made aware of the topics to be discussed, the expected duration of the sessions, and the role of the moderator in facilitating meaningful conversations. The specific demographics and solid cancer disease of the participants are reported in Tables 1, 2 below. The consolidated criteria for reporting qualitative research (COREQ) were closely followed in order to assess the rigor of this qualitative study (Tong et al., 2007).

**TABLE 1:** Specific demographics of cancer focus group participants.

Specific demographics of iCAN participants	*N*	Per cent (%)
**Age**		
- <40	1	2
- 40–50	14	30
- 50–60	10	20
- >60	23	48
**Sex**		
- Male	11	23
- Female	37	77
**Educational level**		
- Primary	16	33
-Secondary	25	52
- Higher-secondary	7	15
**Ethnicity**		
- African	1	2
- Caucasian	47	98
- Asiatic	0	0
**Marital status**		
- Married	36	75
- Single	4	8
-Widow/er	8	17
**Smoking habit**		
- Smoker	9	19
-Ex-smoker	22	46
- Never smoked	17	35

**TABLE 2:** Specific cancer disease and care treatment of cancer focus group participants.

Variable	Category	*N*	Per cent (%)
Cancer type	Breast	23	48
	Oral squamous cell	5	10.5
	Reproductive system	4	8
	Lung	11	23
	prostate	5	10.5
Stage	0	0	0
	I	0	0
	II	0	0
	III	23	48
	IV	25	52
Metastasis	Yes	48	100
	No	0	0
Chemotherapy/ radiotherapy	Yes	48	100
	No	0	0
Surgical treatment	Yes	48	100
	No	0	0

The eligibility criteria were:

• solid cancer patients who have completed or are undergoing medical treatment
• age of patients ≥18 years
• the ability to read and understand informed consent.

The exclusion criteria were:

• pregnancy
• undergoing treatment for anxiety and/or depressive disorders.

Thereafter, patients had a brief interview with a psychologist (MB), who was an expert in the humanization of care. During the interview, an anonymous, multiple-choice questionnaire was proposed and completed by the participants (Table 3) in order to assess needs and critical issues and to select which topic they would like to discuss. Subsequently, the participants were divided into four groups with simple randomization techniques, using web-based simple randomization software (Random.Org, 2022). Table 4 reports the topics selected by the majority of the participants, the preference percentages, and the specialists involved.

**TABLE 3:** Anonymous multiple-choice questionnaire topics.

Topics proposed by patients	Preference percentage (%)
Management of musculoskeletal pain	75%
Management of gastrointestinal issues	25%
Management of nerve pain	30%
Management of cardio-respiratory issues	34%
Emotional and psychological support	73%
Management of ENT issues	43%
Nutritional support	71%
Management of oral issues (mucosa and teeth)	80%
Management of side effects from radio/chemotherapy treatments	70%

**TABLE 4:** Topics chosen by the majority of the participants, preference percentages, and specialists involved.

Selected topics	Preference percentages (%)	Specialist involved
Management of oral issues (mucosa and teeth)	80%	Expert in oral medicine and dental hygiene
Management of musculoskeletal pain	75%	Orthopedic
Emotional and psychological support	73%	Psychologist
Nutritional support	71%	Expert in nutrition science
Management of side effects of radio/chemotherapy treatments	70%	Expert in oral medicine and dental hygiene, orthopedic, psychologist, and expert in nutrition science

### 3.2. Rules governing the iCAN focus group

#### 3.2.1. Setting and timing

Each of the 16 meetings took place in one of reading rooms of the University hospital in Palermo, a large, bright, and welcoming room, with space for a circular arrangement of twelve participants, who were seated without assigned seats. Following the guidance of the moderator (MB), a cognitive behavioral psychotherapist, all patients asked the specialist questions regarding a specific topic. In addition to the moderator, a note-taker was also present, transcribing the order of answers throughout the discussion and the participants’ tone and facial expressions; no video recording was made (Smithson, 2000; Merton and Kendall, 2015). Four live meetings for each group were conducted by the moderator and the specialist, involving patients for a duration of 90 min (Wong, 2008) and the meetings were held 2 days a week from September to December 2019.

#### 3.2.2. Main characteristics of the iCAN focus group

One role of the moderator was to weave together the patients’ ideas, who then posed appropriate questions in plain language. The conversation was conducted in an unintrusive manner, whilst encouraging development of the topic, where possible. Interaction between the patients was encouraged by the moderator, who remained neutral. Thus, the enthusiasm and interest of groups, that is, the group dynamics, were maintained to ensure the active participation of all participants. At the end of each meeting, written feedback was requested from the patients.

#### 3.2.3. Format of the iCAN focus group

The focus group meeting was opened with a welcome from the moderator, who introduced themselves and the specialist, providing a brief overview of the topic of the day. At the beginning of the first meeting, the moderator set out the rules of the setting, explaining the housekeeping rules of the meeting and roles of the iCAN focus group. Thereafter, the moderator asked the members to introduce themselves as part of an ice-breaking activity and to foster relationships among the group members. All the patients were encouraged to speak individually (to avoid confusion and maintain polite group dynamics), emphasizing that all their questions were of equal value with no right or wrong answers (Wong, 2008).

#### 3.2.4. Topics of meetings and key questions

The moderator introduced the discussion in each meeting via randomly asked questions, e.g., *“Has the meaning of your life changed since you received your cancer diagnosis? If so, how*?” and *“What kind of help would you like to receive?”* Thereafter, four specialisms were chosen, such as: orthopedics, nutritional science, dental hygiene, and oral medicine. The moderator and the patients initiated the discussion of each specialism. The interaction was structured by means of a discussion guide (Table 5) through direct questions from patients. There were also comprehensive and definitive answers from the specialists regarding the needs of cancer patients and the knowledge of professionals in improving the quality of life during the treatment period, e.g., *“Did you ever feel that you could not find the strength to cope with treatment?,” “How has your daily life changed because of your bone pain?,” “Can you go on long walks or do one hour of physical activity a day?,” “Do you know the possible side effects from chemotherapy and radiation therapy that may occur on the mucosa of the oral cavity?”* (van der Spek et al., 2013).

**TABLE 5:** Discussion guide.

Topics	Specialist	Key question
Introduction to the discussions with random questions during all meetings by moderator	Psychologist	– Has the meaning of your life changed since your cancer diagnosis? If so, how? – Did you ever feel that you could not find the strength to cope with treatment? If not, how did you cope with your treatment? – What kind of help would you like to receive in the future?
Musculoskeletal issues and pain management	Orthopedic	– How has your daily life changed because of your bone pain? – Can you take long walks or do one hour of physical activity a day? Which remedies do you use to dull the pain? – Do you often use anti-inflammatory medication and/or painkillers?
Nutrition and the cancer patient	Nutrition specialist	– What food do you like eating? – Which foods have you had to give up during your cancer treatment? – Which foods worsen your nausea and/or vomiting?
Oral cavity care during cancer treatment	Oral hygienist	– How often and how do you clean your mouth? – Do you use a mouthwash and floss? – Which foods make chewing or swallowing difficult?
Prevention of mucositis and drug-induced osteonecrosis of the jaw bones	Oral medicine expert	– When did you last have a dental check-up? – Have you had any issues with salivary secretion during your cancer treatment? – Have you experienced pain or developed oral lesions as a result of your cancer treatment? – Do you know the side effects of treatment with anti-resorptive drugs and bisphosphonates? – Do you know the possible side effects from chemotherapy and radiation therapy that may occur on the mucosa of the oral cavity?

#### 3.2.5. Anonymous questionnaire assessment of the meeting

The effectiveness of the meeting was evaluated by means of a questionnaire at its conclusion: the first two questions regarded the conviviality and effectiveness of the meeting, requesting a 5-point response numerical rating scale (van Berckel et al., 2017). The third question questioned whether any change in the format of subsequent meetings should be implemented, while the final question was open-ended, inviting the participants to make suggestions or highlight any critical issues.

## 4. Results

### 4.1. Topics of meetings and key questions

Ninety percent of the participants reported that their lives had changed after receiving their cancer diagnosis, and 60% had been obliged to terminate employment. More than 70% of the participants reported the desire to improve their quality of life, and over 80% reported wanting to discuss issues related to daily living. Eighty percent of participants experienced a feeling of despair due to uncertainty regarding their illness. Indeed, they had difficulty setting goals and planning meaningful activities for the future. Over 35% of participants had to give up their sports and hobbies, and 60% could no longer participate in Sunday walks or social interactions due to metastatic pain.

Painkillers for managing the symptoms of post-cancer treatment pain were often used by 55% of participants. More than 68% of participants reported no longer eating strong-tasting foods due to their cancer treatment, 40% reduced or eliminated any intake of excessively sugary or fatty foods, and more than 60% reported that fried foods, baked cereals, sweets, animal proteins, and foods with strong odors caused nausea and vomiting.

Sixty-five percent of participants maintained their dental hygiene regime at home twice a day, 25% of participants brushed their teeth 3 times a day, with only 5% reporting the absence of maintaining any daily dental hygiene. Toothbrush, mouthwash, and floss were used by 40% of participants, 10% only used a toothbrush, and 50% used toothbrush and mouthwash every day. Seventy per cent of participants reported difficulty swallowing solid foods due to the presence of lesions, and 60% reported that radiotherapy and chemotherapy treatments led to a significant reduction in salivary secretion and that they were obliged to resort to salivary substitutes. Sixty percent of participants reported their last dental private appointment (due to dental issues) as being more than 6 months ago, 30% reported their last appointment as having occurred more than 1 year ago, whilst only 10% reported their last dental private appointment to having been less than 1 month ago. Injuries related to post-cancer treatment pain in the oral cavity were referred to by 65% of participants. Only 25% were aware of the oral adverse effects of bone modifying agents (BMAs), and more than 80% of the participants were unaware of the different side effects caused by radiotherapy and chemotherapy on the oral mucosa.

### 4.2. Anonymous questionnaire assessment of the meeting

The results of the forty-eight anonymous questionnaires are shown in Table 6. With reference to the first question (“*How would you rate the effectiveness of this meeting*?”), 25% of participants answered with a satisfaction rating of “good,” with the remaining 75% indicating a satisfaction value between “very good” and “excellent.” Concerning the second question (“*Was this meeting useful?”*), the authors recorded a satisfaction value of “good” in 14% of participants, a value of “very good” in 35%, and an “excellent” value in 50% of participants. With respect to the third question (“*After this meeting, have your prospects for the future changed?”*) 66.6% responded satisfactorily (*Yes*). The fourth, open-ended question (“*Suggestion or Critical”*) received several suggestions: 33% of the participants suggested an improvement in group communication, 18% suggested using pictures and visuals during the meeting, 35% requested more meetings and 6% suggested a more comfortable setting.

**TABLE 6:** Results of the anonymous questionnaire assessment of the meeting.

Anonymous questionnaire assessment of the meeting		Results
		***N*. of patients (%)**
(1) “How would you rate this effectiveness of this meeting?”	1 (Unacceptable)	0 (0)
	2 (Less than acceptable)	0 (0)
	3 (Good)	12 (25%)
	4 (Very good)	17 (35%)
	5 (Excellent)	19 (40%)
(2) “Was this meeting useful regarding coping in the future?”	1 (Unacceptable)	0 (0)
	2 (Less than acceptable)	0 (0)
	3 (Good)	7 (15%)
	4 (Very good)	17 (35%)
	5 (Excellent)	24 (50%)
(3) “After this meeting, have your prospects for the future changed?”	Yes	32 (67%)
	No	16 (33%)
(4) “Suggestions or comments” (open answers)	Improve communication	16 (33%)
	Use pictures or visuals	9 (19%)
	More meetings	17 (35%)
	More welcoming settings	6 (13%)

## 5. Discussion

Many in the field consider the focus group a useful tool to support not only awareness of a given disease but also to identify patients’ beliefs, which in turn are related to the health risks of behavior types and associated dangers. It includes the duration of group interaction and discussion, both of which are also useful in understanding patients’ experiences of health and health services (Wong, 2008; van der Spek et al., 2013; Merton and Kendall, 2015; Mauceri et al., 2022). There currently exist few studies in the medical literature of many languages which explore the needs, critical issues, and difficulties of cancer patients (Lee and Lee, 2018; Victorson et al., 2019). Moreover, it has been noted that there is a paucity of information relating to patients’ needs in the pre-diagnosis phase, i.e., screening, and in the post-diagnosis phase, i.e., the management stage (Hoesseini et al., 2020). In order to provide support to cancer patients, iCAN focus groups have directed their attention to some of main critical issues topics associated with cancer, as selected by patients. The latter include: emotional needs, pain management, nutrition information, oral health, and dental hygiene.

An appraisal of emotional needs includes psychological distress (depression and anxiety), which is inextricably related to higher cancer-specific mortality and poorer cancer survival rates (Wang et al., 2020). Wang et al. (2020) have suggested that depression and anxiety may have an etiological role and prognostic impact on cancer, although there is potential reverse causality. In addition, the impairment of emotional needs in the cancer pathway also affects the post-diagnosis phase, particularly the loss or return to work. In an observational study Cavanna et al. (2019) have observed similar results to those outlined in this research with job abandonment occurring within 6 months of a cancer diagnosis in 50% of the observed sample. Chen et al. (2021) have emphasized that the development of post-diagnosis emotional rehabilitation, and an occupational counseling programme is necessary to reduce patients’ feelings of hopelessness, helplessness, and health worry. The issue of employment in cancer patients must be carefully considered by the healthcare personnel and institutions with appropriate organizational and regulatory intervention (Chen et al., 2021).

The first pillar of the iCAN focus group was pain, which is often a chronic and disabling condition; it significantly interferes with the functional capacity and quality of life of cancer patients, as reported by Clézardin et al. (2021) and Kapoor et al. (2021). A multidisciplinary approach including surgery, radiation therapy, and medical and behavioral techniques for the management of bone pain is currently best practice in many hospital settings. Many questions have been satisfactorily answered regarding the causes and management, where possible, of cancer bone pain during iCAN discussions. This symptom of cancer can significantly decrease mobility. Many “satisfied” patients underlined how awareness could be considered part of their treatment. For example, the practice of mindfulness techniques seems to be capable of reducing the severity of pain, anxiety, stress, and depression, all of which are related to cancer disease (Grossman et al., 2004; Ngamkham et al., 2019).

The second pillar of the iCAN focus group regarded the oral side effects of chemotherapy and radiation treatments, often resulting in reduced salivary flow, mucositis, and dysphagia, conditions which can cause swallowing difficulties. Consequently, there is a need for the use of salivary substitutes, swallowing rehabilitation exercises, and nutritional support, as also suggested by Kristensen et al. (2020). The latter advocate a multidisciplinary approach to care, including nutritional screening, assessment, and effective intervention regarding diet; all of these have been demonstrated to improve outcomes in terms of nutritional requirements, nutritional status, and the quality of life of cancer patients (Kristensen et al., 2020). Constructive advice was often suggested during iCAN focus group meetings regarding an appropriate diet, which can help reduce the risk of treatment resistance and simultaneously improve the efficacy of cancer treatments (Tajan and Vousden, 2020).

The third pillar of the iCAN focus group concerned home and professional oral health management, with discouraging results having been reported during iCAN focus group meetings. Conflicting information and the education of patients in maintaining their home oral health regime, and dental hygiene is a cause of reduced quality of life for many patients (Yuwanati et al., 2021). Having examined aspects of dental hygiene education and dental evaluation pre-, during and post-cancer, Epstein et al. (2018) demonstrated a general lack of consistency regarding how, when, and from whom oral cancer patients receive their oral health education. In turn, this can contribute to ineffective education, thereby resulting in high levels of patient dissatisfaction with their dental hygiene (Epstein et al., 2018).

The lack of compliance with dental follow-up programmes and the paucity of information provided to patients, regarding the side effects of cancer treatments, emerged during the various iCAN meetings. This highlighted the need to develop personalized and multidisciplinary follow-up programmes, as also advocated by Brands et al. (2021) who have examined ways of optimizing routine follow-up programmes in patients being treated for oral cancer. In supporting patients, the authors created a patient information leaflet with current guidelines regarding oral mucositis, which is the most common side effect of cancer treatments (Elad et al., 2020).

Addressing the innovation introduced within the study, it is imperative to underscore the presence of an acknowledge expert, who is capable of addressing patients’ inquiries and resolving any uncertainties regarding their oncological condition. The authors of this research consider that this innovation offers a significant advancement when compared to conventional methodology. The integration of a dedicated expert into the remit of focus group sessions provides participants with an unparalleled opportunity to glean precise and reliable information. The aim of this approach is to surpass the more typical boundaries, which are often associated with doubts regarding intricate medical tenets and the “deconstruction” of clinical data.

The pivotal role of the specialized expert lies in: providing comprehensive explanations concerning cancer-related nuances, the types of treatment available, potential side effects, and prospects for prudent management. Their participation can foster meaningful dialog, thereby empowering patients to ask questions, express uncertainties, and exchange personal experiences without feeling inhibited. The authors of this research hope that such a pioneering practice may encourage patient engagement in focus groups, thereby increasing the efficacy of the processes involved in focus group participation.

In conclusion, the results outlined in this research demonstrate that the focus groups can have a positive effect in defining patient needs through consciousness-raising. It is essential that patients are informed in supporting and maximizing their general health and quality of life during a course of cancer treatment. The implementation of iCAN focus groups could be effective in addressing this need. The authors wish to highlight the need for patients to recognize themselves in the group and, particularly, the importance of the meeting space in sharing the many daily difficulties of patients. The latter include: uncertainty about their future health, and the emotional discomfort created by illnesses. Furthermore, the feedback collected at the end of the iCAN focus group meetings has facilitated the creation of patient information leaflets, which can assist in meeting the daily oral health needs of cancer patients. (These leaflets can be freely accessed at this link).^1^ The media promotion of health by means of such leaflets and posters can reach a large section of the population. They can also play an important role in disseminating knowledge and skills and encouraging positive changes to societal attitudes toward cancer (Barik et al., 2019).

## 6. Future perspectives

The results of these cancer focus groups have highlighted the importance of counseling and support for cancer patients during their treatment (Zaharias et al., 2018). The authors of this paper hold that cancer focus groups should become a fundamental part of cancer management. They should be not only multidisciplinary but also encompass the idea that cancer patients wish to engage with their treatment.

## 7. Limitations

Inevitably, iCAN meetings have been limited due to the recent SARS-CoV-2 pandemic, and patient access to medical facilities and clinical practice has changed drastically during and to some extent post the pandemic. This has led to the suspension of many cancer focus group meetings. A further disadvantage of focus groups could be the susceptibility to bias because the opinions of the group and individuals can be influenced by dominant participants or the moderator. The requirement for specialized personnel within healthcare facilities is undoubtedly a significant limitation. No recording of discussions was made in this study due to the lack of operators in the project (Tong et al., 2007) and considerations of privacy. The authors of this paper, therefore, wish to specify that there can be diverse and copious topics of interest to cancer patients, and further research is necessary in describing a multidisciplinary approach in curing or mitigating the effects of cancer and caring for the patient.

## 8. Conclusion

Meetings and interviews in cancer focus group have enabled patients to clarify their beliefs and doubts regarding cancer therapy and its implications, especially relating to oral health and dental hygiene. Today it is increasingly important for the patient to comply with planned treatments in the full awareness of suggested healthcare measure. The authors of this study feel that they have been able to move beyond the traditional disease-centered medical approach with this study, thus emphasizing patient involvement. In addition, they have attempted to promote the development of a systemic, integrated, and multidisciplinary approach in which the team, suitably trained, can play a substantial role in the wellbeing of the community. In conclusion, cancer focus group meetings would appear to have facilitated narrative interviewing, improved the doctor-patient relationship, and provide the basis of the humanization of the care process.

## Data availability statement

The original contributions presented in this study are included in the article/supplementary material, further inquiries can be directed to the corresponding author.

## Ethics statement

The studies involving humans were approved by the Comitato Etico Palermo 1 (approval number 4/2015). The studies were conducted in accordance with the local legislation and institutional requirements. The participants provided their written informed consent to participate in this study.

## Author contributions

OD, MB, and GC: conceptualization. GL: methodology and formal analysis. GC and OD: validation. OD, MB, RM, and VP: investigation and data curation. GL and MB: writing original draft preparation. OD: writing review and editing. GC: supervision and project administration. All authors have read and agreed to the published version of the manuscript.

## Footnotes

1
www.policlinico.pa.it/portal/index.php?option=displaypage&Itemid=353&op=page&
